# Insights into the Complex Associations Between MHC Class II DRB Polymorphism and Multiple Gastrointestinal Parasite Infestations in the Striped Mouse

**DOI:** 10.1371/journal.pone.0031820

**Published:** 2012-02-28

**Authors:** Götz Froeschke, Simone Sommer

**Affiliations:** Evolutionary Genetics, Leibniz-Institute for Zoo and Wildlife Research (IZW), Berlin, Germany; Pennsylvania State University College of Medicine, United States of America

## Abstract

Differences in host susceptibility to different parasite types are largely based on the degree of matching between immune genes and parasite antigens. Specifically the variable genes of the major histocompatibility complex (MHC) play a major role in the defence of parasites. However, underlying genetic mechanisms in wild populations are still not well understood because there is a lack of studies which deal with multiple parasite infections and their competition within. To gain insights into these complex associations, we implemented the full record of gastrointestinal nematodes from 439 genotyped individuals of the striped mouse, *Rhabdomys pumilio*. We used two different multivariate approaches to test for associations between MHC class II DRB genotype and multiple nematodes with regard to the main pathogen-driven selection hypotheses maintaining MHC diversity and parasite species-specific co-evolutionary effects. The former includes investigations of a ‘*heterozygote advantage*’, or its specific form a ‘*divergent-allele advantage*’ caused by highly dissimilar alleles as well as possible effects of specific MHC-alleles selected by a ‘*rare allele advantage*’ ( = *negative* ‘*frequency-dependent selection*’). A combination of generalized linear mixed models (GLMMs) and co-inertia (COIA) analyses made it possible to consider multiple parasite species despite the risk of type I errors on the population and on the individual level. We could not find any evidence for a ‘*heterozygote*’ advantage but support for ‘*divergent-allele*’ advantage and infection intensity. In addition, both approaches demonstrated high concordance of positive as well as negative associations between specific MHC alleles and certain parasite species. Furthermore, certain MHC alleles were associated with more than one parasite species, suggesting a many-to-many gene-parasite co-evolution. The most frequent allele *Rhpu*-DRB*38 revealed a pleiotropic effect, involving three nematode species. Our study demonstrates the co-existence of specialist and generalist MHC alleles in terms of parasite detection which may be an important feature in the maintenance of MHC polymorphism.

## Introduction

More than 50% of the known species on this planet are parasites or pathogens of some form [1]. Helminths represent the most prevalent macroparasite group of endoparasites [2] and especially gastrointestinal nematodes can have a large impact on human and animal health [3], [4]. It is known that intestinal worm infections generally cause a strong host immune response e.g. [5]–[7].

Although genetic control of worm burden is likely to be polygenic and it is acknowledged that the immune response is also regulated by interleukin receptor genes [8], [9] recent studies have emphasized the importance of immune genes of the major histocompatibility complex (MHC). The highly polymorphic MHC genes control the immunological self/non-self recognition. MHC molecules bind foreign peptides on the cell surface and present them to T-cells, which then trigger the appropriate immune response [10]. Many studies give evidence that high MHC polymorphism is maintained by pathogen-driven selection either due to the effects of specific MHC-alleles (‘*rare allele advantage hypothesis*’ or ‘*frequency-dependent selection*’, [11]) or an advantage of heterozygote individuals (‘*heterozygote advantage*’, [12]). The ‘heterozygote advantage hypothesis’ presumes that heterozygotes have a higher fitness than homozygotes due to their ability to recognize a wider variety of antigens derived from multiple pathogens. Thus the potential advantage for individuals carrying more than one allele may maintain high numbers of different alleles in populations. Within the heterozygotic genotypes, a more mechanistic explanation suggests that specifically those individuals possessing highly dissimilar MHC alleles potentially bind an even broader range of antigenic peptides then heterozygotes with less dissimilar alleles which may confer a broader immune competence in the case of varying or multiple infestations (‘*divergent allele advantage hypothesis*’, [12]–[15]). However, despite a tremendous effort identifying the relative importance of these mechanisms, the complex dynamics of parasite-host-interactions still remain elusive [16], [17].

In the wild, most animals, including humans, are simultaneously infected with more than one parasite [18]–[20]. Positive and negative associations can occur between parasites mirroring interactions which might cause substantial effects on the parasite load [21], [20]. Especially, macroparasites such as intestinal helminths contain many antigens to which immune responses can be generated. It is estimated that intestinal helminths possess 7,000 to 20,000 protein-encoding genes and even if only the bindings of surface proteins are considered, hosts have many immune targets [22]. Each MHC glycoprotein receptor can bind several hundred different peptides if they have certain sequence characteristics in common [23], [24]. However, despite the high diversity of helminths, immune responses of mammalian hosts seem to vary only relatively little. Generally, binding of helminth antigens to MHC glyoproteins induce a typical CD4+ T helper cell type 2 (Th2) cytokine response [25]–[27]. Therefore it has been suggested that the host immune system has only a limited ability to distinguish among different nematode parasites [25] contradicting the assumption that host pathogen co-evolutionary processes drive genetic diversity. It is argued that classic one-to-one gene parasite co-evolution models do not allow maintenance of diversity because one would expect the fixation of only the latest host allele or parasite strain [28]. By contrast, the existence of a many-to-many gene-parasite co-evolution is expected [29]. If so, multiple specialist and generalist MHC alleles in terms of pathogen detection should co-exist.

So far many MHC studies have presented associations between particular MHC alleles/haplotypes and resistance or susceptibility to single parasite infections (reviewed in [30], [16], e.g. [31]). However, in order to gain deeper insights into the intricate immune reactions and the co-evolutionary processes, it is essential to take into account the interactions between specific MHC alleles within the host species and distinct parasite species within the whole corresponding parasite community. Only then potential antagonistic effects, as well as possible interplays mediated by each parasite species can be detected [32]. Furthermore, it allows the examination of whether specific immune gene variations are associated with multiple parasite species. Up to today only few studies have explored the particular selection pressure exerted by each parasite species in multiple-infected animals ([32], [33], [9]). Hosts with widespread geographical distributions tend to harbour more parasite species than hosts with restricted geographical ranges [34]. To investigate the specificity of associations between MHC alleles/genotypes and different parasite species we therefore chose the striped mouse, *Rhabdomys pumilio,* as our focus species. This rodent is widely distributed in southern Africa and abundant in rural as well as urban areas [35]. Previous studies conducted in *R. pumilio* have already shown evidence for both historical and contemporary balancing as well as directional selection acting on the MHC DRB locus due to parasite pressure [36] (Froeschke and Sommer, unpublished data). These associations are considered as the most direct evidence that parasites work as selective agents to MHC genotypes [37].

The present study was designed to gain a deeper understanding of the genetic bases of host-pathogen co-evolutionary interactions in a multiple-infected host. For this we had the full record of gastrointestinal nematode species recovered from each genetically analysed host-individual available across the entire geographic range of the species, reaching from the Cape region in South Africa up to Northern Namibia [38]. Our specific aim was to investigate possible associations between the MHC class II DRB gene constitution and multiple nematodes a) on the population and b) on the individual level to gain insight into the underlying selection mechanisms and parasite species-specific co-evolutionary effects. For this we applied two multivariate approaches, which allowed us to confound for random factors and at the same time to minimize the impact of type I statistical errors due to multiple testing. We hypothesize that specialist and generalist MHC alleles in terms of pathogen detection are able to co-exist and thus add to the maintenance of MHC polymorphism in the wild.

This is one of the first large-scale-studies on possible correlations between MHC DRB Class II genes and parasites in wild small mammal populations taking into account the whole spectrum of gastrointestinal nematode species. Therefore the results contribute to a deeper understanding of the still poorly understood co-evolutionary dynamics of parasite-host-interactions.

## Results

### Genetic diversity and pathogen-driven selection analyses on the population level

All the results from our microsatellite and MHC genetic diversity analyses can be found in Table 1. Mean microsatellite *D^2^* values varied a lot between populations with the lowest value of 196.18 in population 1 and the highest mean of 524.00 in the population 4. The MHC allelic richness varied between 29.18 at population 1 and 44.86 in population 2. Every population except for the population 4 had a significant observed heterozygosity deficit compared to the expected one. Population 4 showed the lowest null allele frequency. In contrast, the population 3 had the biggest heterozygosity deficit and at the same time the largest proposed null allele frequency (0.176).

**Table 1:** Genetic diversity in seven populations of *R. pumilio*.

		Microsatellites		MHC			
Pop	N	Mean MLH	Mean *D^2^*	Allelic richness	H_obs_/H_exp_	Mean AAdist.	Null allele frequency
1	42	0.974	196.18	29.18	0.67/0.94	0.115	0.133
2	50	1.353	253.82	44.86	0.70/0.97	0.109	0.132
3	65	0.999	266.99	41.56	0.62/0.96	0.103	0.176
4	40	1.012	524.00	33.00	0.85/0.85	0.146	0.001
5	87	1.013	307.96	35.88	0.79/0.96	0.157	0.082
6	115	0.991	356.07	34.06	0.71/0.95	0.133	0.122
7	40	0.976	327.08	44.00	0.78/0.98	0.153	0.059

Genetic diversity in seven populations of *R. pumilio*. Pop = population, N = sample size, MLH = multilocus heterozygosity, *D^2^* = difference in repeat microsatellite units averaged over all loci, H_obs_ = observed heterozygosity, H_exp_ = expected heterozygosity according to Hardy-Weinberg, AAdist = amino acid distance. Allelic richness was corrected for the sample size. Null allele frequency after Dempster et al. (1977). Details on parasite load per population can be found in Froeschke et al. (2010).

We investigated the effects of population genetic diversity on the overall nematode load and all results are listed in Table 2. Neither neutral genetic nor MHC diversity showed significant effects on the nematode load. No support for a heterozygote advantage (nematode prevalence: *P* = 0.46; nematode infection intensity: *P* = 0.99) or divergent-allele advantage (amino acid distance (AAdist): nematode prevalence: *P* = 0.97; nematode infection intensity: *P* = 0.55) or association with MHC allelic richness (nematode prevalence: *P* = 0.95; nematode infection intensity: *P* = 0.28) could be detected on the population level.

**Table 2:** Effects of genetic diversity on nematode load in seven populations of *R. pumilio*.

(a) Prevalence	*ß* ± SE	*T*	*P*
*Model 1*			
Year	0.177±0.278	0.638	0.57
MLH	2.855±2.964	0.963	0.41
*Model 2*			
Year	0.185±0.338	0.545	0.62
*D^2^*	0.001±0.002	0.533	0.63
*Model 3*			
Year	0.087±0.408	0.212	0.85
MHC H_OBS_	2.914±3.479	0.838	0.46
*Model 4*			
Year	0.386±0.238	1.622	0.20
AAdist	0.736±6.545	0.112	0.92
*Model 5*			
Year	0.417±0.242	1.723	0.18
Allelic Richness	−0.006±0.089	−0.067	0.95

(b) Infection intensity (FEC)			
*Model 1*			
Year	−0.403±0.546	−0.738	0.51
MLH	3.828±5.990	0.639	0.57
*Model 2*			
Year	0.147±0.473	0.312	0.78
*D^2^*	−0.002±0.003	−0.703	0.53
*Model 3*			
Year	−0.096±0.603	−0.159	0.88
MHC H_OBS_	−0.045±5.123	−0.009	0.99
*Model 4*			
Year	0.002±0.366	0.004	0.99
AAdist	−5.871±12.190	−0.482	0.66
*Model 5*			
Year	0.210±0.079	0.626	0.58
Allelic Richness	−0.103±0.079	−1.303	0.28

Effects of genetic diversity on nematode load in seven populations of *R. pumilio*, calculated by generalized linear mixed models. Given are the full models for (a) nematode prevalence and (b) nematode infection intensity (FEC). The capture year was included in each model and the population as random factor. ß ± SE stands for the coefficient ± standard error, *t* = t-value, *P* = p significance value.

### Pathogen-driven selection analyses on the individual level

#### (i) Generalized linear mixed models (GLMM)

The two separate generalized linear mixed models (GLMMs), which included either prevalence or infection intensity (based on faecal egg counts, FEC) as response variables from all nematode species combined and MHC genotypes (homozygote, heterozygote) as well as multilocus heterozygosity (MLH) as predictors did not reveal any support for a ‘*heterozygosity advantage*’. There was no support for the hypothesis that MHC heterozygous individuals are less infected than homozygotes (prevalence: *ß* ± SE = −0.189±0.269, t = −0.701, *P* = 0.484). The same applied to MLH (*ß* ± SE = 0.796±0.611, t = 1.304, *P* = 0.193). Also the restriction to the five most common nematodes (*Syphacia obvelata*, *Heligmonina spira*, *Neoheligmonella capensis*, *Trichuris muris* and *Aspiculuris tetraptera*) did not reveal any evidence for a heterozygote advantage (all *P*>0.12).

However, we found some indication for ‘*divergent-allele advantage*’. MHC AADist was a significant explanatory predictor for the overall infection intensities of all nematodes together (*ß* ± SE = 1.651±0.726, t = 3.310, *P* = 0.024), for the Nippostrongylinae (*ß* ± SE = 1.690±0.750, t = 2.253, *P* = 0.025), and for *Aspiculuris tetraptera* (*ß* ± SE = 4.785±2.094, t = 2.285, *P* = 0.023). The neutral *D^2^* did not show any effects (*ß* ± SE = >0.001±>0.001, t = 0.605, *P* = 0.546).

**Figure 1:**
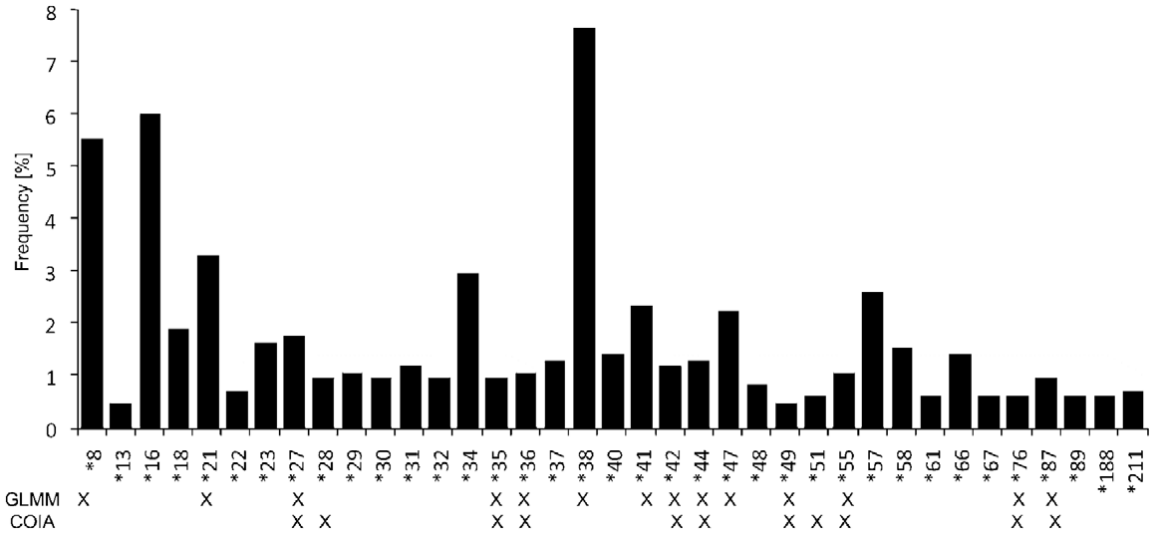
Frequency of the *Rhpu*-DRB alleles. Frequency of the *Rhpu*-DRB alleles (observed in ≥5 individuals). X marks alleles which were detected as associated with a specific nematode species, resulting from generalized linear mixed models (GLMM) and/or co-inertia (COIA) analysis.

Furthermore our GLMMs revealed relationships between specific MHC alleles and prevalence as well as infection intensity (FEC) of the five most prevalent nematodes recorded from *R. pumilio*. Seventeen of the 37 alleles had specific effects either in terms of positive or negative associations towards parasite loads (Table 3, Fig. 1). Whereas no associations between MHC alleles and prevalence of the most abundant nematode *Syphacia obvelata* could be found, the alleles *Rhpu*-DRB*35, *38, *47 and *76 showed a significant effect with an increased infection intensity. Furthermore, positive associations of the two nematode species from the subfamily Nippostrongylinae and the alleles *Rhpu*-DRB*44 and *55 could be revealed for both prevalence and infection intensity. Altogether five alleles were connected with an increased burden of these nematodes. As for *Trichuris muris*, mice which carried the allele *Rhpu*-DRB*38 were significantly less infected than animals without it while the occurrence of alleles *Rhpu*-DRB*42, and *44 was associated with an increased probability of a higher prevalence and infection intensity. Additionally alleles *Rhpu*-DRB*49 and *87 were associated with an elevated infection intensity. The genetic predictors, alleles *Rhpu*-DRB*21, *27, *36 and 41* were significantly associated to the status of infection in *Aspiculuris tetraptera*. Allele *21 was associated with a reduced while the other three alleles were significantly related to a higher prevalence and/or infection intensity.

**Table 3:** Effects of the most abundant *Rhpu*-DRB* alleles on nematode prevalence and infection intensity.

(a) Response variable	Predictor	*ß* ± SE	*t*	*P*	Effect
Prev *_Syphacia_*	Year	1.203±0.360	3.344	<0.001	
	Sex	−0.667±0.277	−2.412	0.016	
Prev _Nippostrongylinae_	*Rhpu* 44	1.608±0.843	1.900	(0.057)	+
	*Rhpu* 55	2.113±1.001	2.111	0.035	+
Prev *_Trichuris_*	*Rhpu* 38	−1.572±0.610	−2.580	0.010	−
	*Rhpu* 42	4.633±1.045	4.434	<0.001	+
	*Rhpu* 44	3.222±0.597	5.401	<0.001	+
	*Rhpu* 55	2.281±0.582	3.920	<0.001	+
Prev *_Aspiculuris_*	Sex	−0.890±0.392	−2.269	0.024	
	*Rhpu* 21	−1.899±0.727	−2.611	0.009	−
	*Rhpu* 36	4.431±1.575	2.814	0.005	+

(b) Response variable					
FEC *_Syphacia_*	Year	0.697±0.284	2.452	0.015	
	Sex	−0.753±0.218	−3.453	<0.001	
	*Rhpu* 35	1.638±0.678	2.417	0.016	+
	*Rhpu* 38	0.692±0.279	2.483	0.013	+
	*Rhpu* 47	1.178±0.466	2.528	0.012	+
	*Rhpu* 76	1.657±0.529	3.135	0.002	+
FEC _Nippostrongylinae_	Year	−0.787±0.190	−4.132	<0.001	
	Sex	−0.575±0.170	−3.375	<0.001	
	*Rhpu* 8	0.953±0.177	5.373	<0.001	+
	*Rhpu* 27	0.763±0.232	3.294	0.001	+
	*Rhpu* 38	0.396±0.169	2.350	0.019	+
	*Rhpu* 44	0.801±0.203	3.948	<0.001	+
	*Rhpu* 55	1.808±0.282	6.411	<0.001	+
FEC *_Trichuris_*	Sex	0.654±0.314	2.085	0.038	
	*Rhpu* 38	−2.781±1.240	−2.243	0.025	−
	*Rhpu* 42	5.837±1.552	3.760	<0.001	+
	*Rhpu* 44	2.831±0.550	5.145	<0.001	+
	*Rhpu* 49	2.366±0.370	6.396	<0.001	+
	*Rhpu* 87	2.153±0.421	5.120	<0.001	+
FEC *_Aspiculuris_*	*Rhpu* 21	−2.024±0.858	−2.358	0.019	−
	*Rhpu* 27	1.530±0.691	2.216	0.027	+
	*Rhpu* 36	5.586±0.706	7.907	<0.001	+
	*Rhpu* 41	2.125±0.929	2.286	0.023	+

Significant effects of the most abundant *Rhpu*-DRB* alleles (frequency ≥5 individuals) on the response variables (a) prevalence and (b) infection intensity (FEC) of the five most prevalent nematode species (*Syphacia obvelata*, Nippostrongylina (*Heligmonina spira*, *Neoheligmonella capensis*) *Trichuris muris* and *Aspiculuris tetraptera*). ß ± SE stands for the coefficient ± standard error, *t* = t-value, *P* = p significance value.

#### (ii) Co-inertia analysis (COIA)

The first two axes of the correspondence analysis, which is based on the genetic table, explained 7.2% of the variance in the data (3.6% F1, 3.6% F2) (factor map not shown) while the axes of the principal component model (PCA), which is based on the FEC table, explained 24.0% of it (12.8% F1, 11.2% F2) (Figure S1). The PCA revealed that the nematode *Syphacia obvelata* was located in the opposite direction to *Aspiculuris tetraptera* and Nematode C. Nematodes A–E are based on egg morphotypes and could not be identified to species level.

**Figure 2:**
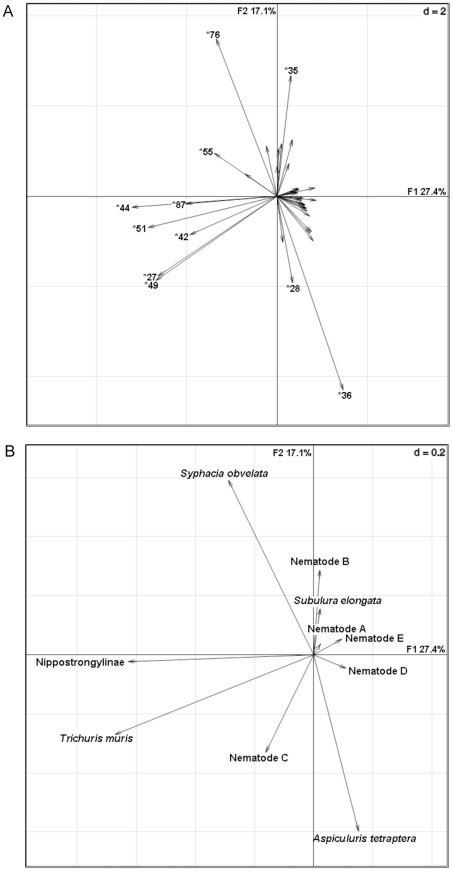
Projection of results of A parasitological and B genetic co-intertia analysis. Projection of results of (A) parasitological and (B) genetic co-inertia analysis from *Rhabdomys pumilio* (n = 432). Variables located in a common direction are positively associated whereas those located in the opposite direction are considered as negatively associated. Variables located close to the centre do not structure the data and are not labelled to improve clarity. Nematode A–E = based on egg morphotypes.

As for the COIA, the first two axes accounted for 44.5% (27.4% F1, 17.1% F2) of the variance shared between genetic and nematode infection intensity matrices. We found no significant overall relationship between the two matrices (Rv-coefficient = 0.060, simulated p = 0.410) but still the co-inertia factor map pointed to associations of certain parasites with the presence of specific MHC alleles (Fig. 2 A, B). The alleles *Rhpu*-DRB *76 and *35 were located in the opposite direction to the alleles *Rhpu*-DRB *36 and *28 on the F2 axes and therefore have antagonist effects. Specifically alleles *Rhpu*-DRB *76 and *36 structured the data at F2. Of note is allele *Rhpu*-DRB *76, which showed a strong association with *S. obvelata* and allele *Rhpu*-DRB *36 which was positively associated with *A. tetraptera*. On the F1 axes mainly the alleles *Rhpu*-DRB *44, *51, *27, *49, *87 and *42 discriminated the data. Alleles *Rhpu*-DRB *44, *87, *51 and *55 showed positive associations with the Nippostrongylinae and alleles *Rhpu*-DRB *42, *49 and *27 with *Trichuris muris.*

The two approaches revealed that 16 out of the 37 MHC alleles (GLMMs: 14; COIA: 11) have effects on the nematode burden (Fig. 1). Nine of the eleven associations (82%) identified by COIA were concordant to those identified by GLMMs.

## Discussion

We applied multivariate approaches to take into account the whole spectrum of gastrointestinal nematode species to advance our understanding of the underlying pathogen-driven selection mechanisms and parasite species-specific co-evolutionary effects on the population and on the individual level using a widely distributed African rodent, *R. pumilio*, as an example.

In our study, no significant effect of neutral microsatellite (MLH and *D^2^*) nor functional MHC genetic diversity and parasite load could be detected on the population level. These results imply that the used microsatellite markers are not affected by parasite infestation, which underlines their neutrality. Yet it is important to remark that seven microsatellite loci only partially describe genome-wide variation [39], [40]. Ongoing studies using next generation sequencing technologies should be based on many more markers randomly scattered throughout the genome to investigate the role of overall genomic variability on parasite resistance.

The lack of associations between MHC genetic diversity and parasite load on the population level in our study adds to the mixed results of former studies. Studies have indicated that if hosts and pathogens share a long-term coevolutionary history, selection through diverse pathogens cause high MHC polymorphism in a species or population, whereas low MHC polymorphism indicates the presence of relaxed pathogenic selection pressure [28] [41] [42].

However, in a contrasting unbalanced situation, i.e. after a recent loss of genetic diversity through, for instance, fragmentation effects, species with low MHC diversity could have lost resistance alleles or other important parts of its adaptive evolutionary potential. This would facilitate an easy spread of pathogens throughout the population, because most individuals share the same resistance genotype [43]. The later scenario has been supported by a recent study conducted by Meyer-Lucht and Sommer [44] which revealed positive associations between MHC allelic richness and nematode load in eight populations of the yellow necked mouse (*Apodemus flavicollis*). MHC heterozygosity in this study had no influence. In our case, maybe diverse interactions of parasites and potential specialist and generalist MHC alleles in terms of different pathogen detection are able to co-exist on the individual level and thus obscure the conformity on the population level.

So far, evidence for heterozygote advantage has rarely been found in wild populations (but see [36], [45], [33]) which might be due to the fact that most studies were restricted to single parasite species. However, the advantages of being able to bind to multiple parasite epitopes may only be detected when multiple parasite-mediated immune insults are prevalent [46], [47]. In our study, this short-coming was ruled out as we had the full record of all gastrointestinal nematode species recovered from each genetically analysed individual and included both complete data sets in our GLMM models to test for a ‘*heterozygote advantage*’ on the population as well as on the individual level. It is known that host gender as well as seasonal changes can have significant effects of parasite infection patterns (e.g. [48]–[50]). By using the individual multivariate GLMM approach we were able to focus on ‘pure’ parasite-driven selection mechanisms because our models allowed us to include confounding factors which could obscure the detection of MHC effects. Therefore sex, trapping season and ‘population’ (which is a synonym for trapping site and a surrogate for the geographical position of each animal) were included as random factors. In our current study based on 424 individuals we found neither support for the ‘*heterozygote advantage hypothesis*’ on the population nor on the individual level. This suggests that heterozygote effects are not likely to play a key role in the maintenance of polymorphism in the MHC of the investigated *R. pumilio* populations. Our current results were in contrast to the observations of a previous study which we conducted on 58 individuals of *R. pumilio* located in the Southern Kalahari [36] where also a lower number of MHC alleles with diverging allele frequencies were observed. Based on recovered egg morphotypes, also the parasite fauna found in mice of the Southern Kalahari was very different (Froeschke & Sommer, unpublished data) compared to the gastrointestinal helminth fauna of the mice identified in this study, which were trapped much further west of the Kalahari. A much higher allelic diversity, which is usually observed in mammals like in our present study, increases the number of potential MHC genotypes and therefore diminishes the chance of detecting homozygotes. Therefore a test for ‘*heterozygote advantage*’ may require a much larger sample size, which becomes practically unfeasible [33]. This implies the difficulties of drawing general conclusions on the importance of MHC heterozygosity on parasitic helminth loads. But again, more in-depth investigations of the role and relative frequency of general and specialist MHC alleles in parasite resistance may provide an advanced picture on the relative importance of the different pathogen-driven MHC selection hypotheses.

Furthermore the possibility of MHC null alleles might obscure our results. As MHC sequences could be amplified from all individuals under study and on the basis of at least two independent PCR and SSCP assays as well as forward and backward sequences, respectively, the obstacle of null alleles is improbable but cannot be ruled out.

However, we found some support for the ‘*divergent allele advantage*’ hypothesis as proposed by Wakeland et al. [15]. The average genetic distance of MHC alleles on the amino acid level within an individual was positively correlated with the overall infection intensities of the total nematode burden. This was significantly influenced by the two Nippostrongylinae species and *Aspiculuris tetraptera.* Naturally it makes sense to give more importance to MHC allele pairs that differ by many amino acids than to those that differ by only a few. Also Lenz et al. [51] found support for the ‘*divergent allele advantage*’ hypothesis in the MHC IIB alleles of the Borneo Long-tailed giant rat (*Leopoldamys sabanus*).

In order to investigate the effects of specific MHC alleles in parasite resistance we used two separate multivariate analysis approaches (GLMM, COIA) as we wanted to take into account a) possible important co-founding factors and b) to control and discuss possible type I errors associated with multiple comparisons. GLMMs are increasingly used to model multivariate nested data, temporal and spatial correlation structures in count data or binomial data [52]. The more explanatory variables in the model, the higher the risk of collinearity, which makes it necessary to test predictors in separate models. In our study we fitted a GLMM for the five most abundant nematode species which amounted to more than 82% of all helminth infections [38], thus increasing the risk of type I errors due to multiple testing. COIA provides a less detailed but more holistic vision of possible associations between MHC constitutions and parasite load because it does not confound for random factors as the GLMMs. Apart from that it is robust to correlation between variables, can be used with all types of variables [53] and has recently been successfully applied to similar MHC-parasitological datasets ([54], [32], [9]).

Both multivariate analyses revealed significant relationships between specific MHC alleles and parasite load on the individual level. The majority of these alleles were positively associated with a high parasite load. The missing overall significant relationship in COIA between genetic and parasitological matrices is probably caused by the co-founding factors as they were sex, population and year which could not be included here, unlike in the GLMMs. Specifically nine MHC alleles showed positive associations to certain parasite species in our GLMM analysis, which also pointed to the same direction in our COIA (Fig. 1). We found 11 alleles in our GLMMs (Table 2 B), which were significantly associated with intensity in only one model. Due to multiple testing one should apply a more stringent significance level but maybe valuable information would get lost. We believe that a second multivariate approach can add reassurance to cases like for example allele *Rhpu 35.* After applying a stringent significance level (like e.g. Bonferroni) it would not be considered anymore as associated to the infection intensity of *S. obvelata.* The COIA nevertheless confirms its positive association and therefore it should be regarded as one of the more ‘specialized’ MHC alleles. Only allele *Rhpu 41* might be considered as a statistical artifact and has to be treated with extra caution because it only occurs in one GLMM, its p-value cannot withstand a Bonferroni correction and also our COIA does not show any possible association. In general, our two applied different statistical methods show high concordance in the results in terms of alleles which featured specific associations with parasite burden (Fig. 1).

‘Disadvantageous’ MHC alleles, positively associated with infection, have also already been detected in several other studies (e.g. [36], [54]–[58]. This is usually interpreted as a support for the ‘rare allele advantage’ or ‘frequency-dependent selection’ [11] hypothesis proposing a co-evolutionary arms race between the pathogen and the host with dynamic and reciprocal cycling of the frequency of specific ‘protective’ MHC alleles and certain parasites. Hughes and Nei [46] claim that under natural conditions there is no good biological reason to believe that a previously favoured allele will become rare again, given the fact that it can bind several hundred peptides. But recent studies emphasize the importance of antagonistic effects of MHC alleles in pathogen resistance which might explain on one hand their persistance but on the other hand also strong frequency shifts in a population. Loiseau et al. [58] found in their study on malaria infections in house sparrows (*Passer domesticus*) that an MHC class I allele was associated with an increased risk in being infected with *Plasmodium*, but at the same time connected to a severe reduction in the risk to harbour a *Haemoproteus* strain. Those antagonistic effects for different parasite species may contribute to the maintenance of alleles in populations, which appear to be disadvantageous at first. We also found support for this conclusion in our study: GLMMs showed that the overall most abundant allele, *Rhpu*-DRB*38 was associated with elevated infestation rates of *S. obvelata* and Nippostrongylinae. At the same time its occurrence was negatively correlated to the burden of *T. muris*. Up to now, such an antagonistic, pleiotropic effect of an allele has rarely been shown in a natural animal host-parasite system before. A comparison of the amino acid sequences revealed that allele *Rhpu*-DRB *38 differs from all other alleles which showed associations by having a glutamatic acid residue at the antigen binding site (ABS) position 53 and a leucine residual directly neighbouring at position 54. The other alleles carried valine, alanine, arginine or glutamine at position 53 and valine at position 54 instead. Position 53 is a positively selected site in *R. pumilio* and also an antigen-binding site in humans [59]. Therefore it can be regarded as functionally important. Substitutions in the antigen binding site of an MHC molecule might e.g. reduce or support the binding of specific antigens to the MHC molecule which in turn might influence the whole immune response (summarized by [60], [61]). Further pleiotropic effects were revealed in the principal component analysis as well as the co-inertia analysis. Both showed oppositions between *S. obvelata* and *A. tetraptera* which co-occur in the same habitats and thus host specimen [38]. Often different helminth species in the same host specimen cause competition for nutrients and space [62], [63]. However, in *R. pumilio* and in studies conducted with laboratory mice and wild populations of *Mus musculus*, *S. obvelata* was recovered from the cecum while *A. tetraptera* was found in the small intestines [38], [64]. Therefore we attribute the discovered opposition in *R. pumilio* to an antagonist role of MHC allele-specific resistance and susceptibility to both nematodes.

More and more studies suggest that multiple parasites are required to drive MHC polymorphism [47], [28], [29] and a many-to-many instead of a one-to-one gene-parasite co-evolution is proposed (see discussion in [29]). Tellier and Brown [65] applied a simplified model and concluded that stable polymorphism is most likely to be detected in systems with strongly polycyclic diseases with high autoinfection. Our study supports this assumption of a many-to-many gene-parasite co-evolution because GLMMs revealed that the alleles *Rhpu*-DRB *27, *38 and *44 showed associations with more than one nematode species. At the same time each nematode species was associated with more than one MHC allele. An elevated infection intensity of *Syphacia obvelata,* the most prevalent nematode in our study, was explained by positive associations with the alleles *Rhpu*-DRB*35, *38, *47 and *76. Allele *38 is the most frequent one in our study (overall frequency: 7.74%, Fig. 1). Also the parasite load of other nematode species, such as *Heligmonina spira*, *Neoheligmonella capensis*, *Aspiculuris tetraptera* as well as the whipworm *Trichuris muris* showed positive as well as negative associations with different MHC alleles. These observations were also supported by the COIA. Also the co-inertia factor map presented specific alleles with positive associations to more than one parasite species (like e.g. *Rhpu*-DRB*44 with *T. muris* and the Nippostrongylinae) and therefore going along with a proposed many-to-many gene-parasite co-evolution.

But we must keep in mind that though the MHC is of immense importance in parasite defence, also other genes are involved in the immune response cascade. A recent study by Schwensow et al. [9] showed for instance complex associations of the expression levels of TGF-ß, IL-10, IL-4 and IL-2 with species may depress the host's intestinal immune response, which may cause advantages for other parasites and their life traits (reviewed in [21], [66]). Furthermore, certain parasite are suspected to be immune suppressors [67]. Only one key parasite species might be sufficient to create the overall positive host immuno-mediated association structure if other species are affected by its immunosuppressive capacity [68].

To conclude, our large-scale study showed that certain MHC alleles were associated with more than one parasite species and vice versa. Specialist and generalist MHC alleles in terms of different pathogen detection are able to co-exist, thus favouring a many-to-many gene-parasite co-evolutionary prospect. Pleiotropic effects and further complex interactions must be considered when dealing with multi-infected host species in the wild and parasites as a driver for MHC class II DRB polymorphism.

We propose for future studies, that it is not only important to draw the attention on MHC gene diversity but also on the allele specificity to gain a more accurate picture. For the future it will be mandatory to characterize parasites antigens to a similar extent as their hosts immune genes to fully understand the process of host-parasite co-evolution.

## Materials and Methods

### Study sites and sample collection

The study has been conducted in accordance with the recommendations for care and use of animals approved by Ministry of Environment and Tourism, Namibia (permit no 853/2004 and 1065/2006), the Northern Cape Nature Conservation Service, South Africa (permit no 0592/04 and 0133/06) and the Cape Nature Department of the Western Cape, South Africa (permit no 001-202-00021 and AAA004-00029-0035).

We captured 470 individuals at seven different sites, each considered as a population of the striped mouse (*Rhabdomys pumilio*), across a large geographic range, reaching from the Cape Floristic Region in the south of South Africa up to the north of Namibia. A more detailed description about the site-specific meteorological variables can be found in Froeschke et al. [38]. Samples were taken twice, between November 2004–March 2005 and then again June–August 2006. Animals were marked with ear tags but no recaptures were observed between the capture sessions. We used standardized grid systems, consisting of Sherman traps 15 m apart from one another. Traps were baited with a peanut butter-, apple- and oats mixture. To avoid overheating of trapped animals we only opened traps from dusk until dawn. Throughout our study, only one mouse was caught per trap allowing us to allocate faeces collected from the trap to a single individual from which also a tissue sample from the ear was taken for genetic investigations. All samples were stored in 70% ethanol for later parasitological and genetical analyses. In order to limit age effects on parasite load only adult animals (n = 439) weighing ≥32 g [69] were considered for further studies. During the second capture session a subsample of 161 adult individuals were euthanized with isoflurane (Forene®, Abbott GmbH, Germany) and the gastrointestinal tracts (stomach, small intestine and caecum) were dissected out and stored in 70% ethanol [38].

### Parasite Screening

We took advantage of previously published nematode data for this study and a detailed description of parasite screening and distribution can be found in Froeschke et al. [38] (also see Table S1). Briefly, to measure the gastrointestinal nematode prevalence and infection intensity of the 439 individuals, we applied faecal egg counts (FEC, number of eggs per gram faeces) using a McMaster floatation technique modified by Meyer-Lucht and Sommer [56]. A standardised volume of 200 mg faeces was used per animal. To recover and identify adult worms we screened all 161 dissected gastrointestinal tracts and used published species descriptions (a list can be supplied by the authors), scanning electron microscopy and personal communications with leading experts (see acknowledgements) in the affiliated field. A comparison of the worms that were recorded in the gastrointestinal tract and faecal material revealed that all of the highly abundant egg morphotypes could be linked to the adult nematodes found in the gastrointestinal tract [38]. Only the eggs from the two nematodes *Heligmonina spira* and *Neoheligmonella capensis* could not be distinguished because of their similar shape and size and therefore they were grouped together to their subfamily Nippostrongylinae.

### Molecular Techniques

The molecular techniques have been described previously [36] and all nucleotide sequences from the MHC DRB exon II region can be found at GenBank (Accession numbers AY928313, AY928314, AY928318–AY928320, AY928324–AY928327, AY928329, GU332030–GU332268). In short, DNA was extracted from ear tissue using the DNeasy Tissue Kit (Qiagen, Hilden, Germany) and we used the primers JS1 and JS2, which amplify a 171-bp fragment [70] of MHC class II DRB exon2. The investigated region contains all the alleged functionally important antigen binding sites, derived from humans (ABS; [58]), and evidence for positive selection has been already shown in Froeschke and Sommer [36]. We applied the single stranded conformation polymorphism (SSCP) method to identify allelic diversity (following the manufacturer's protocol,; ETC Elektrophoresetechnik, Kirchentellinsfurt, Germany). SSCP bands were subsequently cut out of the gel and re-amplified. No more than two alleles per individual could be detected, suggesting that only one MHC locus was amplified. Cycle sequencing was performed with an Applied Biosystems automated sequencer model 3130, using a dye terminator sequencing kit (Applied Biosystems, Forster City, CA). Forward and backward sequences from each newly discovered allele were thereby taken from two separate PCR and SSCP assays, respectively, to confirm their allelic make-up. Sequences were edited and aligned with the software MEGA 4 [71]. Furthermore all individuals were genotyped at seven microsatellite markers. Details on the microsatellites, heterozygosity and null allele analyses are presented elsewhere (Froeschke and Sommer, in review).

### Genetic diversity analysis

To measure the overall genetic neutral diversity per population we used MLH (multilocus heterozygosity, [72] and mean microsatellite *D^2^* (difference in repeat units, averaged over all loci, [73].

MHC genetic diversity was described using the observed heterozygosity and mean- and individual amino acid distance (AADist) as well as the allelic richness. Observed and expected heterozygosity for the MHC per population was calculated with the software Arlequin 3.0 [74]. AADist was calculated with the software MEGA 4 [71]. Because the observed number of alleles in a sample is highly dependent on the number of sampled individuals, we calculated the allelic richness corrected for different sample sizes by using a rarefaction index implemented in FSTAT [75]. Thereby, the expected number of alleles in each population is calculated for the number of individuals present in the smallest population. Frequency of null alleles was estimated with the algorithm presented in Dempster et al. [76] implemented in the program FREENA [77].

### Pathogen-driven selection analyses on the population level

To investigate associations between multiple nematode infestation and the gene constitutions considering both type of markers on the population level we used multivariate generalized linear mixed models (GLMM). Models were fitted for overall nematode prevalence and overall mean nematode infection intensity. The models for prevalence were calculated using a binomial error distribution and logit link function. In the models for mean infection intensity we applied Gaussian error distribution with an identity link function [44].

Due to the small number of seven populations and to avoid collinearity the five predictors of genetic diversity (microsatellite MLH, microsatellite *D^2^*, MHC heterozygosity, MHC AAdist and MHC allelic richness) were included in separate but otherwise identical models. Capture season [year] was added as a further predictor in each model. Because each trapping site and thus each population was characterized by a site-specific precipitation pattern, which had a big influence on the parasite load [38] we included ‘population’ as a random factor in our models.

### Pathogen-driven selection analyses on the individual level

In order to test for possible associations and interactions between MHC alleles and specific nematodes as well as finding support for parasite-driven selection mechanisms on the individual level, we applied two different approaches and used (i) GLMM and (ii) co-inertia analysis (COIA). In all cases, the error structure of the parasite response variables was not normally distributed. To avoid collinearity in our GLMMs, which investigate the association between the individual genetic constitution and parasite load, we tested all predictors for correlations and, if necessary, included them in separate, otherwise identical models.

To test for ‘*heterozygote advantage*’ and ‘*divergent allele advantage*’ we took (a) the prevalence (presence/absence of a parasite) and (b) the infection intensity (FEC) data of all parasites species together, as well as of the five most abundant nematodes *Syphacia obvelata*, *Heligmonina spira* and *Neoheligmonella capensis* (combined to subfamily Nippostrongylinae), *Trichuris muris* and *Aspiculuris tetraptera* separately, as response variables. These five parasite species amounted to more than 82% of all helminth infections [38]. Heterozygote host individuals and animals with a higher allele divergence should be able to recognize a broader spectrum of parasites and thus potential lower prevalence and FEC rates would be interpreted as an advantage. The models for prevalence were calculated using a binomial error distribution and logit link function. In the models for infection intensity we applied a quasipoisson error distribution with a loglink function, which accounts for overdispersion in these data [78]. As predictors for ‘*heterozygote advantage*’ we included the MHC genotype (homo- or heterozygote) and microsatellite MLH for each individual as fixed factors in our model as well as sex and capture season [year]. To consider extra sources of variation in variances through the influences of different populations and accordingly geographical position of each individual, we added ‘population’ as a random factor.

Afterwards we applied a similar model as described above with the covariates AADist and *D^2^* as fixed predictors to investigate a possible ‘*divergent allele advantage*’.

To examine possible association between specific MHC alleles (‘*rare allele advantage*’) and parasite species, we continued with the five most abundant nematode species. Again the models for prevalence were calculated using a binomial error distribution and logit link function, and for the models for infection intensity we applied a quasipoisson error distribution with a loglink function. As predictors we included the presence/absence of the 37 alleles observed in more than five individuals (Fig. 1) as fixed factors as well as sex and capture season [year]. ‘Population’ from each individual was included again as a random factor.

For our GLMMs we had the required data (individual parasite, MHC and microsatellite data, sex) of 424 individuals available. Models were validated by the examination of the plots of residuals against fitted values and checked for heteroscedasticity and outliers. Predictors with extreme standard errors (<100 000, p = 1) were excluded from the specific model [78]. They were caused by the fact that some of the scarce nematode species and alleles did not co-occur in all populations. All models were conducted in R [79] and implemented the function glmmPQL [80], using the package MASS [81] and NLME [82].

For the second approach to estimate possible positive or negative associations between specific MHC alleles and helminth burden we performed a co-inertia analysis (COIA) with the complete dataset. Here we pooled our data based on the assumption that individual allele-parasite co-evolution should hold across populations [83].

A COIA is a multivariate method for coupling two tables with the only constraint that the sites are weighted in the same way for each table [53]. This method has been used in the past mainly for ecological data and only recently applied for genetic – parasite interaction studies [32], [83], [9], [84]. First we conducted a correspondence analysis (COA) with the genetic presence/absence table of the 37 MHC alleles included in the analysis. As a second step we performed a principal component analysis (PCA) with the parasitological matrix, including the infection intensity (FEC) of eleven of thirteen previously recovered gastrointestinal nematodes [38]. The nematodes *Streptopharagus sudanensis* and *Trichostrongylus probulurus* were excluded from the analysis since we only had FEC data from one and two individuals available, respectively. We then performed a co-inertia (COIA) analysis to link the COA and PCA analysed matrices and assessed associations between MHC alleles and nematode infection intensity visually on factorial maps. The significance of correlation between genetic and parasitological matrices was measured with the Rv-coefficient. Significance between both matrixes was assessed with a permutation test, which compares 1000 randomly generated data sets with the real data set. COA, PCA and COIA analyses were performed with the ade4TkGUI package. All statistical tests were conducted in R [79].

## Supporting Information

Figure S1 **Projection of results of parasitological principal component analysis.** Projection of results of parasitological principal component analysis from *Rhabdomys pumilio* (n = 432). Variables located in a common direction are positively associated whereas those located in the opposite direction are considered as negatively associated. Variables located close to the centre do not structure the data and are not labelled to improve clarity. Nematode A–E = based on egg morphotypes.
(TIF)

Table S1 **Nematode load per population.** Nematode infestation rate [%], mean species richness, abundance [log no. of worms] and mean infection intensity [logEPG] per population ± S.E.
(DOC)
