# Supplementary figures and images for: Insights into the Complex Associations Between MHC Class II DRB Polymorphism and Multiple Gastrointestinal Parasite Infestations in the Striped Mouse
Source: PLoS One. 2012 Feb 28;7(2):e31820. doi: 10.1371/journal.pone.0031820 (PMC3289624; doi:10.1371/journal.pone.0031820)

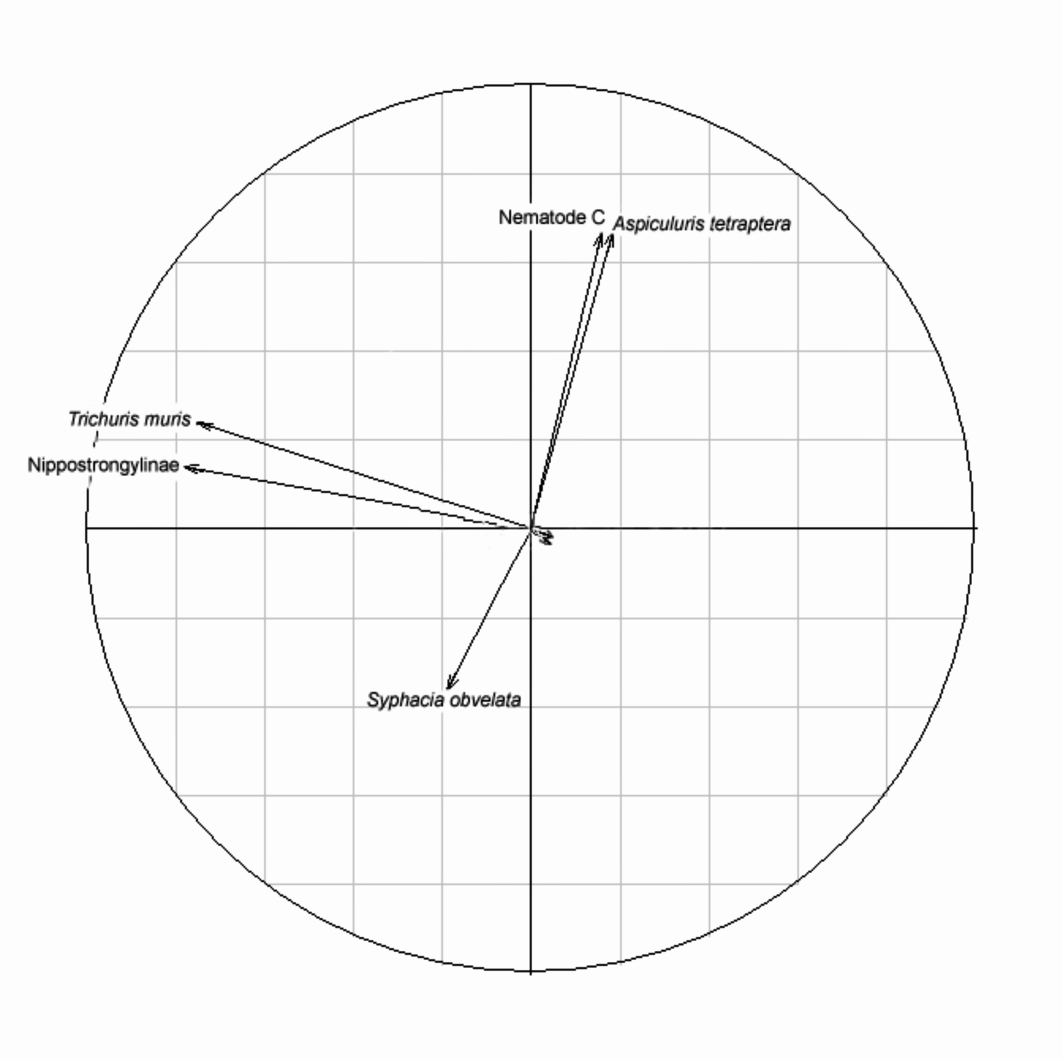

Supplement: Figure S1 — Projection of results of parasitological principal component analysis. Projection of results of parasitological principal component analysis from Rhabdomys pumilio (n = 432). Variables located in a common direction are positively associated whereas those located in the opposite direction are considered as negatively associated. Variables located close to the centre do not structure the data and are not labelled to improve clarity. Nematode A–E = based on egg morphotypes. (TIF) [file pone.0031820.s001.tif]
