# Supplementary material for: Insights into the Complex Associations Between MHC Class II DRB Polymorphism and Multiple Gastrointestinal Parasite Infestations in the Striped Mouse
Source: PLoS One. 2012 Feb 28;7(2):e31820. doi: 10.1371/journal.pone.0031820 (PMC3289624; doi:10.1371/journal.pone.0031820)
Supplement: Table S1 — Nematode load per population. Nematode infestation rate [%], mean species richness, abundance [log no. of worms] and mean infection intensity [logEPG] per population ± S.E. (DOC) [file pone.0031820.s002.doc]

**Table S1: Nematode load per population**

|  | South Africa | | | Namibia | | | |
| --- | --- | --- | --- | --- | --- | --- | --- |
| Population | **1** | **2** | **3** | **4** | **5** | **6** | **7** |
| Infestation [%] | 97.6 | 85.7 | 37.5 | 22.5 | 31.3 | 25.8 | 41.5 |
| Mean species richness | 2.40 ± 0.25 | 1.75 ± 0.28 | 0.65 ± 0.17 | 0.27 ± 0.12 | 0.40 ± 0.11 | 0.33 ± 0.09 | 0.47 ± 0.19 |
| Mean abundance [log no. of worms] | 1.85 ± 0.22 | 1.59 ± 0.21 | 0.45 ± 0.12 | 0.20 ± 0.10 | 0.33 ± 0.10 | 0.44 ± 0.13 | 0.34 ± 0.15 |
| Mean infection intensity [logEPG] | 3.69 ± 0.14 | 2.45 ± 0.18 | 0.99 ± 0.17 | 0.48 ± 0.15 | 0.65 ± 0.11 | 0.55 ± 0.09 | 0.95 ± 0.18 |

Table S1: Nematode infestation rate [%], mean species richness, abundance [log no. of worms] and mean infection intensity [logEPG] per population ± S.E.
